# Electrical current through individual pairs of phosphorus donor atoms and silicon dangling bonds

**DOI:** 10.1038/srep18531

**Published:** 2016-01-13

**Authors:** K. Ambal, P. Rahe, A. Payne, J. Slinkman, C. C. Williams, C. Boehme

**Affiliations:** 1 Department of Physics and Astronomy, University of Utah, Salt Lake City, UT 84112.; 2 RFSOI Technology Development, IBM Microelectronics, Essex Junction, VT 05452.

## Abstract

Nuclear spins of phosphorus [P] donor atoms in crystalline silicon are among the most coherent qubits found in nature. For their utilization in scalable quantum computers, distinct donor electron wavefunctions must be controlled and probed through electrical coupling by application of either highly localized electric fields or spin-selective currents. Due to the strong modulation of the P-donor wavefunction by the silicon lattice, such electrical coupling requires atomic spatial accuracy. Here, the spatially controlled application of electrical current through individual pairs of phosphorus donor electron states in crystalline silicon and silicon dangling bond states at the crystalline silicon (100) surface is demonstrated using a high‐resolution scanning probe microscope operated under ultra‐high vacuum and at a temperature of 4.3K. The observed pairs of electron states display qualitatively reproducible current-voltage characteristics with a monotonous increase and intermediate current plateaus.

Progressively longer lasting quantum coherence of impurity nuclear spins in solid state environments has been demonstrated in recent years, with coherence times on the order of an hour observed for phosphorus (P) donors in crystalline silicon(c-Si)^1^ reiterating their excellent suitability for quantum information applications^2^. While progress on the development of individual readout schemes for these silicon based spin qubits has been equally swift^3^,^4^,^5^,^6^, the selective electric addressability of individual qubits in qubit arrays with lattice site precision is ultimately needed, not only for the control of interactions between qubits via electric fields^2^, but also for selective readout using spin-selection rules^7^. Thus, due to the strong modulation of P qubit states by the c-Si lattice^8^, establishing such controllable electrical contact to individual P-donor states requires techniques that allow for an atomic-scale positioning of individual electronic probe states in the proximities of qubits. Scanning probe techniques like atomic force microscopy (AFM) and scanning tunneling microscopy (STM) both provide this precision. The latter is based on electric contact to surface and interface states^9^ and resolves surface morphologies by keeping a probe current constant through regulation of the probe-to-surface distance. In the following, we report the application of a hybrid scanning probe microscopy technique based on a combination of non-contact AFM and STM, and the so-called conduction AFM (c-AFM)^10^,^11^ for the study of charge conduction through P impurity donors coupled to silicon dangling bonds (DBs). When the lateral resolution of the scanning probe permits, images of electronic states emerge (P-donor and DBs)^8^,^12^,^13^ that are endpoints of current percolation paths. Such observations cannot be made with standard scanning probe techniques like STM or AFM individually. However, the spatially highly resolved identification of individual phosphorus donor states and the addressability of these states with lattice site precision are necessary steps towards the utilization of phosphorus qubits. This demonstration will also enable alternative concepts for atomic-scale resolution single-spin detection as recently developed by Payne *et al*.^14^ where single electron spin states are detected using spin-dependent tunneling between two paramagnetic states.

The experiments presented here are illustrated in Fig. 1(a) which shows a schematic of the conduction AFM setup consisting of an Omicron Nanotechnology Oxford Instruments LT STM/AFM system using a quartz tuning fork in qPlus configuration^15^ with a clean platinum (Pt) probe tip attached^16^. The tuning fork oscillates at its nominal frequency *f*_0_ with constant amplitude (∼1 nm) under UHV conditions (∼1 × 10^−10^ mBar) whereby the tip-sample interaction leads to a frequency shift Δ*f* used as the topography feedback signal. In contrast to optically detected AFM, using a quartz-tuning fork sensor allows for AFM measurements in the absence of light and thus, photo-charge carriers. The (100) oriented silicon substrate is doped with phosphorus with a dark resistivity of 0.08–0.01 ohm-cm at room temperature and a lower, but still significant dark conductance at 4.3 K due to wave function overlap of the neutral P donor states caused by high dopant concentrations ([P] ∼ 10^17^ cm^−3^ to 10^18^ cm^−3^, see sample preparation in the methods section). We investigate pristine as well as oxide-covered (100) surfaces. For the experiments, a positive DC bias is applied to the tip with respect to the back contact of the substrate. The tip is then brought in proximity of the sample surface while the interaction between the Pt probe and the surface is measured by observation of Δ*f*. The height feedback controller for the qPlus sensor uses the measured gap-dependent frequency shift to control the height of the probe (constant frequency shift) during imaging, with the assumption that each point of the surface provides an equal interaction with the probe tip. Local carrier conduction occurs from sample to Pt tip via tunneling. Both, the tip and sample are in thermal contact with a liquid He^4^ reservoir maintaining a stable temperature of 4.3K.

## Results

### Detection of localized phosphorus donor states

**Figure 1:**
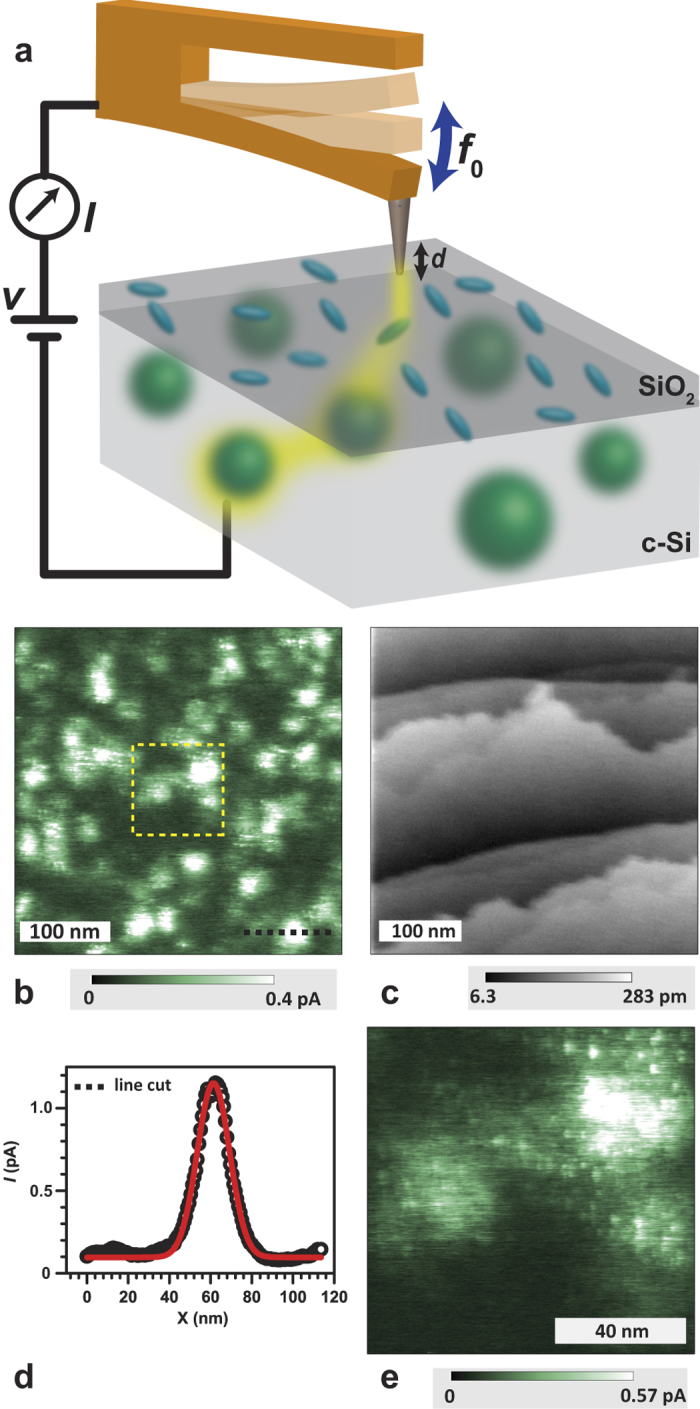
(**a**) Schematic of the low-temperature current imaging experiment conducted on a phosphorus doped silicon substrate. A quartz tuning fork (qPlus sensor) with a Pt tip attached oscillates at *f*_0_ ~ 30 kHz. When a bias voltage *V* is applied to the probe, electrons tunnel from highly localized silicon dangling bond (blue ellipsoids) into the Pt tip. Due to high density of P dopants (indicated by green spheres), the substrate allows for percolation (path indicated by yellow halo) of charges through the bulk. Thus, recharging of the emptied dangling bond state through recombination is possible from a nearby P atom if the donor-dangling bond proximity permits. (**b**) Current map of a P doped, flash cleaned c-Si substrate without any silicon dioxide at 4.3 K in darkness. The tip-bias voltage applied for this measurement is 1.3 V. The bright patches represent the spatial distribution of charge percolation endpoints indicating electronic states consistent with the localization and density of P donor atoms. (**c**) AFM topography image taken simultaneously using the interaction between surface and probe. The individual step edges are resolved. Note the absence of correlation between the current map and the surface topography. We also note that this AFM topography image displays a very weak, albeit recognizable ghost image in the upper right corner of the imaged area. (**d**) Line profile of one patch as indicated by the dotted black line in (**b**). The FWHM of the Gaussian fit (red) of this patch is ~18 nm, corresponding to approximately 3 Bohr diameters of the P donor wave function. (**e**) High resolution image of the area indicated by the yellow box in (**b**) using identical condition of (**b**). A distinct, seemingly random fine structure for some of the patches attributed to the P donors is visible.

The image displayed in Fig. 1(b) represents the conduction AFM detected surface current distribution (current map) of a pristine, atomically clean silicon sample in darkness. The image reveals several ∼20 nm to 30 nm large areas (patches) in which localized current maxima are observed. We have calculated the maximal detectable tunneling distance (see Supplemental Information and ref. 17) from the probe tip to phosphorus states in the sample to be approximately three Bohr radii (the Bohr radius of the s-shaped P donor is ∼3 nm^8^,^18^). We find that both the diameter (FWHM) of these patches, as well as their observed areal density (∼10^11^ cm^−2^ corresponding to ∼10^17^ cm^−3^ with ∼3 Bohr radii accessible probe depth), indicate that the bright regions in the image are likely be caused by the randomly distributed P donor atoms near the silicon surface. As discussed below, the varying physical dimension and brightness of the patches can be attributed to the varied depths of the P donors.

In order to corroborate that the bright patches seen of Fig. 1(b) are due to electronic states rather than surface structure, a surface topography image was measured simultaneously with the data in Fig. 1(b), by recording the probe tip height while maintaining a constant frequency shift. The result of this measurement is displayed in Fig. 1(c). The image reveals a clean surface with monatomic step edges due to a slight miscut of the silicon sample wafer during the fabrication process. A comparison of panels (b) and (c) in Fig. 1 reveals that there is no recognizable correlation between the two images recorded in the same area at the same time. Thus, the features observed in the conduction AFM image must be attributed to localized electronic states.

Figure 1(d) displays a line cut (black line) through one of the patches shown in Fig. 1(b). The peak current is about 1pA with respect to the current pre-amplifier baseline with ∼18 nm full width of half maximum (FWHM). The half current radius of the patches are therefore within approximately three Bohr diameters of the P donor state^8^,^18^, which further supports the hypothesis that the identified patches are due to P donor atoms. Figure 1(e) shows a high-resolution current image taken within the area marked by the yellow box in Fig. 1(b). It resolves some highly localized fine structure of the charge percolation paths within the 10–15 nm broader patches. Two possible explanations are consistent with this observation: (i) the fine structure is due to the crystal periodicity which modulates the electronic wave function of the donor electron^8^ and thus, the electric current through this state. (ii) It is due to silicon surface states (e.g. DB states) which exist at very high densities at the pristine silicon surface for which electronic transitions from P donors are well known^7^.

**Figure 2:**
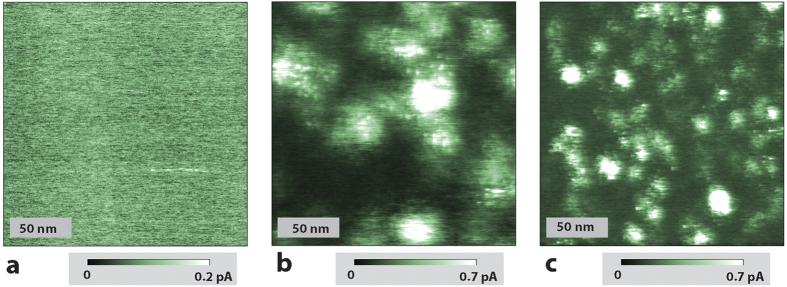
Conduction AFM images of c-Si samples with different P surface and bulk concentrations prepared by ion implantation. The tip-bias voltage applied for each measurement is (**a**) 1.1 V, (**b**) 1.3 V and (**c**) 1.2 V. (**a**) [P] = 5 × 10^14^ cm^−3^. No recognizable current maxima with significance above the noise level are observed. (**b**) [P] = 3 × 10^17^ cm^−3^. Current maxima with areal density of about ~4 × 10^10^ patches/cm^−2^ are observed. (**c**) [P] = 5 × 10^18^ cm^−3^. Current maxima with a high areal density ~2 × 10^11^ patches/cm^−2^ are observed. The observed densities of the patches fluctuate within a 12% range for different locations of the same sample, consistent with stochastic fluctuations expected from the given samples sizes. Note that the maximum current of ∼1pA in (**b**) and ∼5pA in (**c**) exceed the displayed current range. The current range was chosen in order to provide optimal contrast.

In order to test the assignment of the c-AFM observed current “patches” to P donor atoms, conduction AFM images were acquired on 3 samples with different doping concentration prepared as discussed in the methods section below with nominal surface concentrations of [P] = 5 × 10^14^ cm^−3^, 3 × 10^17^ cm^−3^ and 5 × 10^18^ cm^−3^ respectively. Each sample was cleaned and prepared identically. Conduction AFM measurements were then acquired under nominally identical conditions to those discussed above. The results of these experiments are shown in Fig. 2. They reveal that for the two higher dopant density samples displayed in panels (b) and (c), the phosphorus concentrations of [P] = 3 × 10^17^ cm^−3^ and 5 × 10^18^ cm^−3^ correlate well with the areal patch densities of ~4 × 10^10^ cm^−2^ and ~2 × 10^11^ cm^−2^, respectively. Each of these patch densities was obtained from manual counts executed on several conduction AFM images made on the same sample. The counts revealed a fluctuation of 12% between different images on the same samples. We attribute the absence of detectable current for the lowest P-concentration sample ([P] = 5 × 10^14^ cm^−3^) to the lower P-donor density of this sample. This indicates that the density is too small to sustain an extended charge percolation path through the localized donor states to the back contact of the sample. Higher densities are required because all experiments were conducted significantly below the donor ionization temperature (∼30 K) and in darkness where photo-charge carriers are absent. In essence, at a temperature of T = 4.3 K, the more weakly doped c-Si is essentially an insulator and conduction AFM is therefore not possible.

We note that the apparent smaller patch size seen in Fig. 2 for [P] = 5 × 10^18^ cm^−3^ is caused by the small distances between the donor states that drop below natural patch sizes observed at low donor densities (with sizes discussed above). Under this high-[P] condition, the observed patch size will be governed by the donor distance, i.e. the measured patch radius is then given by the distance of the current minimum between the two neighboring patch centers. In essence, the observed patch radius will become half of the nearest neighbor distance as long as the color scale of the image provides enough dynamic range to display the local current minima between adjacent atoms at high densities.

In order to corroborate the findings presented above, the experiments were repeatedly conducted at different sample areas as well as for identically prepared silicon wafers with equal dopant concentrations (see supporting information). For all these experiments, simultaneous conduction AFM and surface topology images were acquired. All these measurements confirmed the occurrence of the fine structured patches, the absence of correlation between the c-AFM recorded patch structure and the surface topography, as well as the presence of a correlation between the patch density and the P dopant concentration. We therefore conclude that these patches are indeed representations of electric current through individual phosphorus donor states.

### Detection of surface dangling bond states

**Figure 3:**
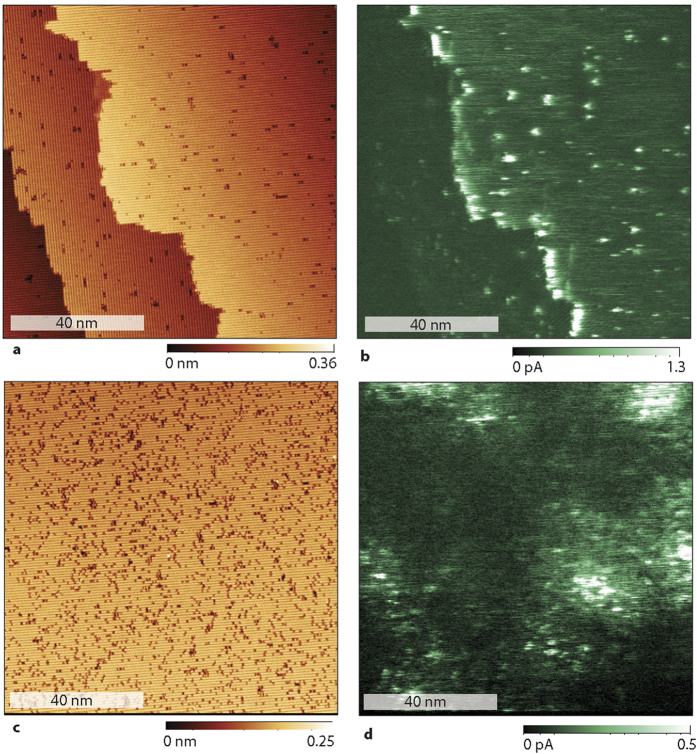
STM images and conduction AFM images of a surface of a P-doped c-Si crystal right after a flash anneal (a,b) and after one day (c,d). Initially, the surface has very few defects apart from step edges (**a**). After a day, the surface has more defects present (**c**). (**b**) A dark conduction AFM image taken at the same location and shortly after image (**a**) at a tip-bias voltage of 1.2 V. It shows that current maxima occur only at a few point-like defects which exist at the surface an at step edges. (**c**) After a day, a higher defect density has developed and now the P-donor patches appear in the dark conduction AFM image (**d**).

In order to investigate the nature of the patch fine structures, STM and c-AFM images were taken on (100) c-Si samples with concentrations of [P] ∼ 10^17^ cm^−3^ to ∼10^18^ cm^−3^ right after flash anneal took place (no oxide) and then again about a day later after a native oxide has been formed. An STM image of the surface taken right after anneal is shown in Fig. 3(a). This data set was acquired at a temperature *T* = 4.3 K, with sample illumination that provided free carrier conductivity to the back contact, and a 20pA current set point with a tip voltage of 2V. The image shows that the surface is fully reconstructed with some defects (dark spots). Figure 3(b) is a conduction AFM image taken in darkness at the same location. It is notable that directly after annealing, the conductance is typically high at the step-edges and other intrinsic morphological defects [dark spots in the STM image in (a)] of the surface. It is also notable that current percolation takes place predominantly through surface defects. Nevertheless, there are also many point defects at the surface [dark spots in the STM image in (a)] where current percolation is weak, proving that some surface defects allow for currents to propagate while others don’t. In consideration of the general increase of current known to exist throughout the patch-like regions defined by the presence of P donor states, the observation of surface defects with and without charge percolation strongly suggests that electric current requires the presence of both, a surface state as well as an underlying donor state. If a surface defect is not connected to a donor state underneath or if a donor state is close to the surface but not to a surface state, there will be no current percolation. We note that for the sample used for the measurements in Fig. 3(a,b), the overall defect density at the surface is so low that their correlation to patch-like structures attributed to the donor atom is not obvious in the conduction AFM images. In contrast, when these STM and c-AFM experiments are repeated a day later on the same sample, with the same tip, and under the same experimental conditions, the results (shown in panels (c) and (d)) differ significantly. The STM measurement (c) now shows a reconstructed surface that has a much higher surface point defect density^19^. The conduction AFM image taken on the same area as the image in panel (c) shows that the conductivity patches attributed to phosphorus donor atoms have now appeared. As seen in both panels (c) and (d), the surface defects are randomly distributed across the surface, yet only those defects electrically connected to a nearby P donor state become visible. Thus, the data in Fig. 3 shows that the small, highly localized states are responsible for the fine structure within the larger current patches, but also for the electrical access to donor atoms in c-Si substrate. Without these highly localized defects, tunneling from the tip to the donor atoms is unlikely. While silicon dangling bonds are expected to exist at homogeneous densities throughout the observed sample areas, current through the highly localized states is observed only when these states connect to an adjacent P donor, which in turn is connected to other P donors which form the percolation paths that provides the observed current. We therefore conclude that electrical current from P donor atoms to the cantilever tip requires the intermediate involvement of these highly localized interface defects.

This realization also corroborates that the above discussed, fine-structured current patches observed with conduction AFM do not represent P donor wave functions; instead, they are indicative of surface defect clusters in close proximity of P donor states underneath the surface. Thus, while the size and the shape of these patches are likely correlated to the P donor wave-function, this correlation is convoluted with surface defect distributions. The charge percolation maps revealed by the data presented are strongly dependent on the probed electronic states and thus, the geometric nature of their wavefunctions but they are nevertheless not directly equitable to the three-dimensional projections of the wavefunctions of the involved states onto the observed two-dimensional data. We note again that the qualitative reproducibility of the experiments shown in Fig. 3 was shown repeatedly with various samples and scanning probes tips.

**Figure 4:**
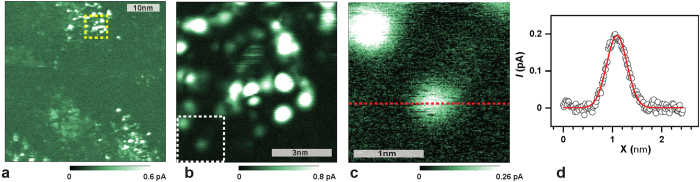
(**a**) Current map obtained from the P doped c-Si substrate at a tip-bias of 1.0 V in darkness at 4.3 K after a thin SiO_2_ film was grown. Large (>20 nm) patches surrounded by low-current regions still exist, yet the fine structure of these patches is significantly more isolated. Overall, the measured current densities are lower as seen in (**b**), which represents an current map with higher scanning resolution obtained on the subarea of (**a**) marked by the yellow square. (**c**) Image zoomed into the subarea marked by a white square in (**b**). (**d**) Plot of the current marked in (**c**) by the red line as a function of lateral position. The displayed individual current maximum has a full width at half maximum of about 6 Å.

Next, the nature of the fine structure within the higher conductivity patches was investigated. In order to determine whether it is caused by silicon DBs, samples with similar P dopant densities but varying surface DB densities were prepared through growth of thin oxide layers. (See methods section for details.) Low-temperature grown (<500 °C) silicon/ silicon-dioxide (Si/SiO_2_) interfaces can exhibit very high DB densities (>10^12^ cm^−2^) which include both crystalline silicon interface states (so-called P_b_ centers) as well as oxygen vacancy defects in the amorphous oxide layer (so-called E’ centers^20^,^21^,^22^). The average separation between those states is therefore only a few nanometers (<10 nm). Figure 4 displays the imaging results on such an oxidized surface (2–3 Å thickness^23^) under otherwise identical conditions as for the measurements described by Fig. 1. Panel (a) confirms that, similar to the image in Fig. 1(e), patch-like regions with FWHM of the order of 10nm to 15nm exist within which higher current densities are observed, while outside of these regions, the current is small. Also, similar to Figs 1(e), and 4(a) displays a distinct fine structure, yet in contrast to Fig. 1(e), the fine structure consists of well-separated, randomly distributed, highly localized current maxima. Figure 4(b,c) display conduction AFM scans of sub-areas of (a) and (b), respectively, with increasing resolution. Note the data set in (b) is not a magnification of the data in (a) but from a separate measurement in the same sub-area. In order to determine the size of this local current maximum, we have determined its full width at half maximum (6 Å) using a linecut taken from the data in (c), as indicated by the red dotted line and shown in Fig. 4(d). Note that the current variation in this localized region is approximately 200 fA.

The width of the localized current maximum seen in Fig. 4(c,d) is determined by either the effective tunneling radius of the probe tip or the actual size of the observed electronic state. Given that all features seen in Fig. 4(b–d) exhibit approximately the same localization, we conclude that it is due to the resolution limit set by the tunneling radius of the tip. This implies that the observed electronic states could be more localized than the widths seen in the image. We therefore believe that these states could be due to silicon DBs at the c-Si surface. Figure 4(a) shows that the highly localized current maxima are only detected within the much larger, tens of nm-sized, patch-like structures attributed above to P donors atoms. Between these patches, larger areas exist where no local current maxima are observed. This observation suggests that charge percolation for the observed data sets and under the given bias conditions, occurs preferably through pairs of P donors and the surface states (e.g. the DB states) rather than directly from the probe tip to the phosphorus donors. For the applied positive tip bias, this is indeed consistent with the well-investigated spin-dependent P/DB transitions^7^,^24^,^25^,^26^,^27^.

### The current-voltage characteristics of P-DB pairs

**Figure 5:**
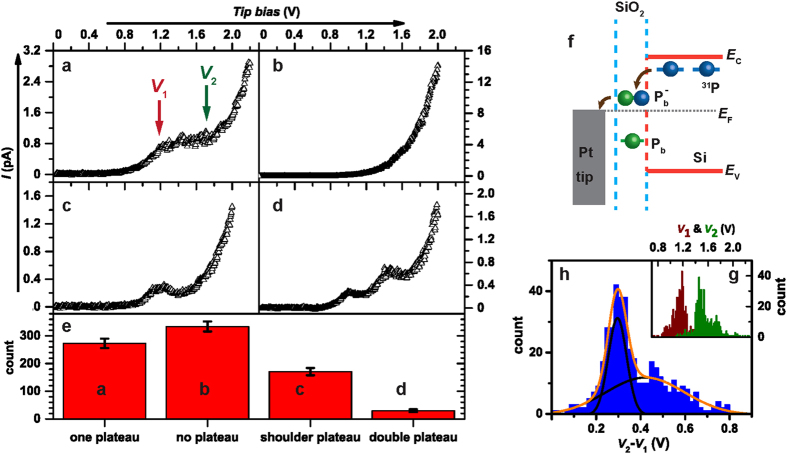
(**a**–**d**) display samples of four qualitatively different types of I–V curves of more than 800 measured different I–V curves acquired on SiO_2_ covered Si(100) surfaces. (**a**) I–V curve with a single flat plateau region; (**b**) I–V curve without plateau as expected from a macroscopic silicon to platinum Schottky diode; (**c**) I–V curve with “tilted” plateau which consists of a local maximum followed by a local minimum; (**d**) I–V curve with double plateau. (**e**) Bar diagrams indicating the actual number of qualitatively distinct I–V curves shown in (**a**) to (**d**) based on the acquired data. The error bars represent the square root of the actual number. (**f**) Energy diagram of the doped silicon sample and tip including donor state and the interface dangling bond state (P_b_ center). (**g**) Histograms of the plateau-onset (brown) and -end (green) voltages of the 415 plateaus from data sets of I–V curves that display a single flat or tilted plateau similar to the data seen in (**a**,**c**). (**h**) Histogram (blue data) displaying the plateau widths of the given data sets (difference between plateau-onset and –end voltage of each curve) as well as the fit (orange line) with a two Gaussian functions (black lines). The good agreement indicates that there are at least two qualitatively different types of highly localized interface states responsible for the local current maxima at the surface. The two Gaussian functions are centered at 300(5)  mV and 420(19) mV.

In order to corroborate the findings presented above and in particular the hypothesis that the charge percolation for the observed conduction AFM images is caused by P-DB transitions, we have repeated the imaging and identified hundreds of locations with highly localized current maxima (a few more images are displayed in the supporting information). At each of these states, the conductive probe was positioned at the location of current maximum and the current was monitored while the probe Fermi-level was lowered through an increase of the probe bias voltage. Examples for the current-voltage (I–V) characteristics obtained at different tip positions, resulting from this procedure, are shown in Fig. 5(a–d). Some of these I–V curves exhibit one (a) or two (d) distinct plateaus on which the current is only weakly bias dependent while others display a bias dependence similar to a macroscopic diode (b)^28^,^29^ or a plateau with negative slope (c)^30^,^31^. All of the more than 800 I–V curves fall qualitatively within one of these four groups.

Figure 5(e) displays the numbers of curves with I–V characteristics of each of the four different cases shown in Fig. 5(a–d). 334 I–V curves show Schottky diode-like characteristics due to tip induced band bending (TIBB) and 273 I–V curves show a single flat plateau. Given the random localization and energies of Si/SiO_2_ interface states (see Fig. 5(f)), many hypotheses explaining the four observed I–V curves can be invoked. For instance, the rare double plateau characteristics could be caused by multiple localized defects being in proximity of the charge percolation path. The single-plateau functions (both with and without negative slope) could be accounted for transitions between the P donor and silicon surface dangling bonds (so-called P_b_ states). The band diagram illustrating the P/P_b_ transitions, as developed from electrically detected magnetic resonance spectroscopy^7^,^24^,^32^,^33^, is shown in Fig. 5(f). According to this model, the plateau occurs when the Fermi level *E*_f_ of the probe is between the energy of the doubly occupied, negatively charged P_b_^−^ state and the single occupied neutral P_b_ state. Little charge transfer will occur at low bias when the *E*_f_ is above the P_b_^−^ level because direct tunneling from P donors into the tip is not likely. In contrast, when *E*_f_ drops below singly occupied P_b_ ground state at high bias, an increasing current passes through the states. Similar hypothesis that are also based on existing P/P_b_ recombination models^7^,^24^,^32^,^33^ and the electric field sensitivity of the donor wave function [P] can be invoked in order to explain the single plateau I–V-behavior with negative slope seen in Fig. 5(c) where quantitative differences between the individual transitions for flat plateau IV functions and negatively sloped I–V functions can account for the qualitatively different behavior. Specifically, one can invoke the P/P_b_-transition as the origin for both, the flat plateau and the shoulder plateau. In both cases, the plateau appears when the P/P_b_-transition becomes the bottleneck for the overall current. While this P/P_b_-transition probability would be entirely bias independent for the case of flat plateaus, there would be a negative rate dependence on the bias for the shoulder plateaus. Such a weak but non-negligible bias dependence is conceivable to appear for certain dangling bond to phosphorous donor geometries due to either local tip induced band bending or due to bias-induced change of the donor wave function (due to the Stark effect^2^), that can lead to a small but significant modulation of the donor-dangling bond exchange interaction.

## Discussion

We have quantitatively scrutinized the attribution of the single-flat-plateau I–V characteristics to conduction through P/P_b_ pairs by examining the widths of the current plateaus for flat plateaus as represented by the I–V function displayed in Fig. 5(a). Literature reports of P_b_ centers at (100) surfaces indicate that the Coulomb repulsion induced correlation energies responsible for the splitting of the singly occupied, neutral P_b_ state and the doubly occupied negatively charged P_b_^−^ state are different for two different types of P_b_ centers, the P_b0_ as well as the P_b1_^20^,^34^. Moreover, due to the inherent disorder of the amorphous silicon dioxide layer in the direct environment of P_b_ centers, correlation energies are strongly distributed^20^,^34^,^35^. Thus, if the single-plateau I–V curves are due to P/P_b_ transitions, the plateau-width distribution must reveal both the presence of two distinct defect types as well as their respective average values. Figure 5(g) (inset) displays a histogram of the plateau-onset and -end bias voltages obtained from the 415 I–V curves observed with single flat plateaus and plateaus with negative slope for which we could determine clear onset and end bias voltages, sorted in 0.02V bins. The difference between the plateau offset and end voltage for each curve as obtained from the data in Fig. 5(g) is displayed in the histogram of Fig. 5(h), also with 0.02 V bin size. For the limited number of available counts, the data reveal an excellent agreement with a double Gaussian fit function, represented by the orange data line. The good agreement with a bimodal distribution supports the expected presence of two types of states. The Gaussian distributions are centered at 300(5) mV and 420(19) mV, in good agreement with capacitance-voltage spectroscopy results of the Si(100)/SiO_2_ interface which shows two types of electronic states^34^,^35^ with electron correlation energies of ~350 meV and ~550 meV. Thus, we attribute the locations where current maxima reveal I–V curves with single, flat plateaus to the presence of highly localized P_b1_ or P_b0_ dangling bonds in proximity of a P donor atom.

In conclusion, the application of low-temperature, high-resolution current imaging under dark conditions to surfaces of strongly P doped c-Si with thin oxide surfaces has allowed us to image charge conduction through individual pairs of P donor and highly localized surface silicon dangling bond (P_b0_ and P_b1_) states, as verified by spatially resolved imaging and energetic considerations. Hence, a method to electrically contact individual pairs of P donor and P_b_ interface states on an atomic length scale is demonstrated, which could serve as a selective address- and readout-technique for individual P donor qubits in c-Si.

## Methods

### Conduction AFM

For conduction AFM, the tip-sample interaction is kept constant using the frequency shift *∆f* of the qPlus sensor as the topography feedback signal during scanning the probe laterally over the surface area. While the tip height obtained from this procedure resolves surface morphology, an electric current through electronic states in the substrate within the tunneling range does not resemble morphological information. Instead, for the samples investigated herein it reveals an image of charge conduction paths which arrive at the sample surface^36^,^37^,^38^. Depending on the scanning probe resolution, the measured tunneling currents can reveal images of the electronic states (the wavefunction) at the conduction path of electronic states closest to the scanning probe. While STM is typically based on tunneling currents in the pA to nA range, these measurements reveal average currents in the low fA range. Due to the frequency-shift feedback-controlled probe positioning, mapping the localized current paths with lateral resolutions in the sub-nanometer range is possible. For the measurements of the I-V characteristics at specific locations, the height control feedback was switched off. This is necessary in order to maintain a constant gap between the tip and sample during the measurement since the bias-dependent electrostatic force would cause the height feedback to withdraw the tip, thereby modify the tip-sample separation. Due to the low drift and creep conditions given at 4.3K, the tip-sample distance is kept constant with high precision.

### Probe preparation

The probes used in this study were provided by Rocky Mountain Nanotechnology, LCC. They were fabricated from a solid Pt wire, exhibiting a 20nm tip radius. The tips were glued to one arm of the tuning fork using conductive glue and placed inside the UHV chamber of the scanning probe system. While the qPlus sensor is under stable oscillation, STM measurements were performed at 77K on atomically cleaned, 7 × 7 reconstructed Si (111) surfaces prepared using standard flash-annealed procedure^37^, to verify the tip conductivity and sharpness allowing for atomic resolution. When a stable conductive tip was confirmed in this process, the scanning probe setup was cooled to liquid ^4^He temperature with the sample being inserted in the microscope. The conduction AFM images were then acquired the following day after the setup had reached thermal equilibrium.

### Sample preparation

The substrates used in this study were single-side polished, PRIME grade, Cz grown c-Si wafers with (100) orientation. The wafers were uniformly doped with P yielding room temperature resistivity between 0.08–0.01 ohm-cm for the data presented in the main text. Different densities were used for the concentration control experiments. The wafers were cut into 10 × 1.2 mm pieces and each piece was pre-cleaned with a standard cleaning procedure including a 20 min ultra-sonic bath in acetone followed by 20 min ultra-sonic bath in IPA, a DI water rinse and blow drying with pure compressed N_2_. All pre-cleaning was done outside the UHV chamber under atmospheric conditions. After pre-cleaning, the sample was clamped onto an Omicron direct-heating molybdenum sample plate and placed inside the scanning probe system. At UHV pressure (<1 × 10^−10^ mBar), the sample was subjected to the main cleaning step involving a thermal annealing. First, the samples were kept for 2 h at 550 °C on a heating stage inside the UHV chamber. Thereafter, a constant DC current (∼0.8 to 1.0 A) was passed through the sample for about 12 hours, increasing the sample temperature to ∼400 to 600 °C, and followed by a flash anneal during which the DC current inside the sample was increased from 0–4.2 A in 0.5 A steps at intervals of 5 s. At 4.2A, the constant current was maintained for 20–30 s before rapid thermal cooling was initiated. The flash process was repeated at least three times, a procedure during which the native oxide at the surface was subsequently removed from the surface and a reconstructed atomically clean flat surface^39^,^40^,^41^ was left behind.

For the native oxide growth, a c-Si (100) substrate was first flash-cleaned as described above and then exposed to ultrahigh-purity compressed oxygen that had been humidified by passing through a water bubbler at 80 °C. The sample surface was then exposed inside the load lock at ambient pressure with the resulting moist oxygen for 2hrs at room temperature, a process in which a very thin ∼2–3 Å native oxide was grown on top of the cleaned silicon surface^23^,^42^.

Silicon samples with different dopant concentrations were made from (100) oriented p-type wafers ([B] = 5 × 10^14^ cm^−3^) by ion implantation with P atoms at 25 keV with an implantation dose of 10^13^, 10^14^ and 10^15^ cm^−2^, respectively. After the ion implantation, a 100 nm epitaxially grown layer was deposited at 550 °C for 10 min followed by a spike anneal at 1050 °C for 0.1 s at 100% Ar ambient and 30 min anneal at 550 °C at 100% N_2_ ambient. Using this technique, three samples with different lower surface concentrations were fabricated which, due to the implanted P layer, still provided the good conductivity needed for the c-AFM experiments. These samples were fabricated and supplied by the Development Laboratory at IBM Microelectronics in Essex Junction, VT.

## Additional Information

**How to cite this article**: Ambal, K. *et al*. Electrical current through individual pairs of phosphorus donor atoms and silicon dangling bonds. *Sci. Rep*. **6**, 18531; doi: 10.1038/srep18531 (2016).

## Supplementary Material

Supplementary Information
