# Supplementary material for: Electrical current through individual pairs of phosphorus donor atoms and silicon dangling bonds
Source: Sci Rep. 2016 Jan 13;6:18531. doi: 10.1038/srep18531 (PMC4725375; doi:10.1038/srep18531)
Supplement: Supplementary Information [file srep18531-s1.pdf]

Electrical coupling to individual pairs of phosphorus donor atoms and silicon dangling bonds

- Supplementary Information –

K. Ambal[1], P. Rahe[1,2], A. Payne[1], J. Slinkman[3], C. C. Williams[1]*, and C. Boehme[1]**

[1]Department of Physics and Astronomy, University of Utah, Salt Lake City, UT 84112

[2]now at: Department of Physics and Astronomy, University of Nottingham, University Park, Nottingham NG7 2RD, UK

[3]RFSOI Technology Development, IBM Microelectronics, Essex Junction, VT 05452

1. Control experiments for the identification of conductivity "patches" as the P donor states

Additional STM images in the region shown by Fig. #1(c), with a 10pA current set point and 2V DC tip-sample bias, are shown in Fig. #S1 (d) to (f), together with a series of corresponding conduction AFM images in identical areas. Figure #S1 (a) and (b) are the same images as shown in Fig. #1 (b), (e) respectively. Panels (b), (d), (e), and (f) of Fig. #S1 display additional data sets. Panels (b), (c), (e), and (f) of Fig. #S1 were recorded on the areas marked by the boxes in (a) and (b), which provide images with successively smaller scale for both the conduction AFM as well as the STM measurements. The data confirms again that there is no recognizable correlation between the STM topography map and the distribution of bright patches in the conduction AFM images (top row). The data also shows that the observed surface is pristine (terraces are observed) and that an atomic scale structure of the conduction AFM current distribution exists which is indicative of either the influence of the crystalline silicon lattice or the presence of highly localized surface defects, e.g. silicon dangling bond states.

We repeated the experiments described by Fig. #S1 and performed STM and conduction AFM experiments various times at various places of the given sample as well as different samples obtained from the same wafer. Figure #S2 (a) and (d) are the conduction AFM and STM images already shown in Fig. #S1 (b) and (e), respectively. These data sets are here compared with conduction AFM and STM images shown in and Fig. #S2 (b), (c), (e), and (f) obtained on different samples at different measurement days that were nominally prepared under the same conditions and from the same silicon wafer. The images were acquired under nominally identical measurement conditions. The STM images (lower row) show that all three measurements reveal different surface point defect densities. While we did not further study the nature of these point defects as well as conditions which favor or suppress their generation, a comparison of the observed densities as well as their increase over time with data of previous UHV studies of c-Si (100)-(2x1) reconstructed surfaces suggests that these are due to water adsorbates [SS1]. We note that the point defects observed here are of different nature than the silicon dangling bond states at Si/SiO_2 interfaces discussed in detail in the main text because the areal densities

of those silicon dangling bond states does not change with time under nominally identical UHV conditions. In contrast, the AFM conduction images (upper) reveal that the larger current patch sizes and spatial distribution are approximately the same. The AFM conduction images also reveal fine structures within these patches. The AFM and STM images show that the terrace steps in the reconstructed surface do not correlate with the location of the large patches. This is consistent with the hypothesis that the patches are caused by P donors.

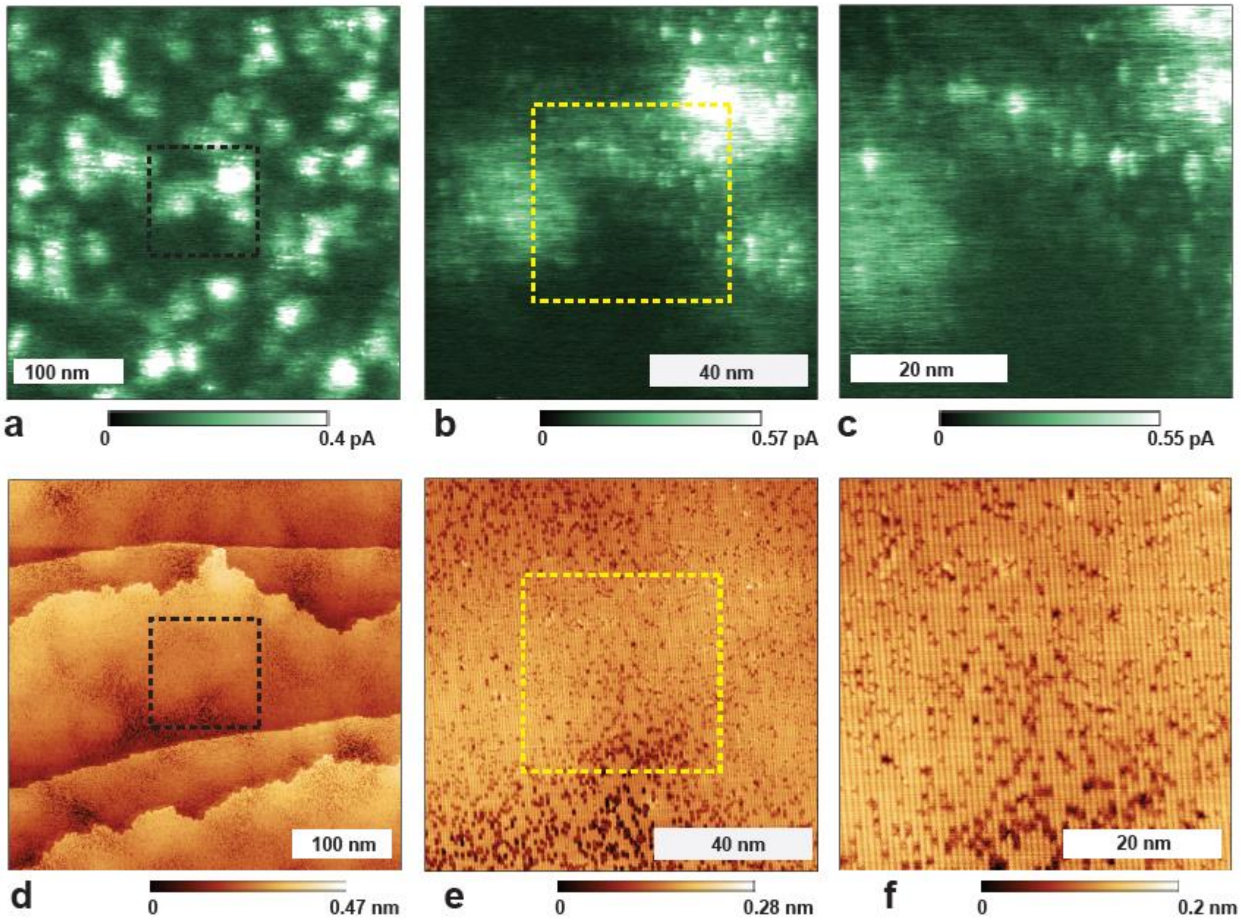

Figure #S1, (a) to (c): Conduction AFM images of c-Si surfaces acquired in darkness at 4.3K. Applied tip voltage is 1.3V for panels (a) to (c). (d) to (f): STM topography images acquired under illumination at 2V tip voltage, corresponding to the areas shown in (a) to (c), respectively. The broad (~30nm) 'patches' seen in (a) to (c) do not correlate with the STM topography of the surfaces displayed in (d) to (f), respectively. (b), (c), (e) and (f) are the high resolution images taken in the respective black and yellow box regions shown in (a), (b), (d) and (e) respectively.

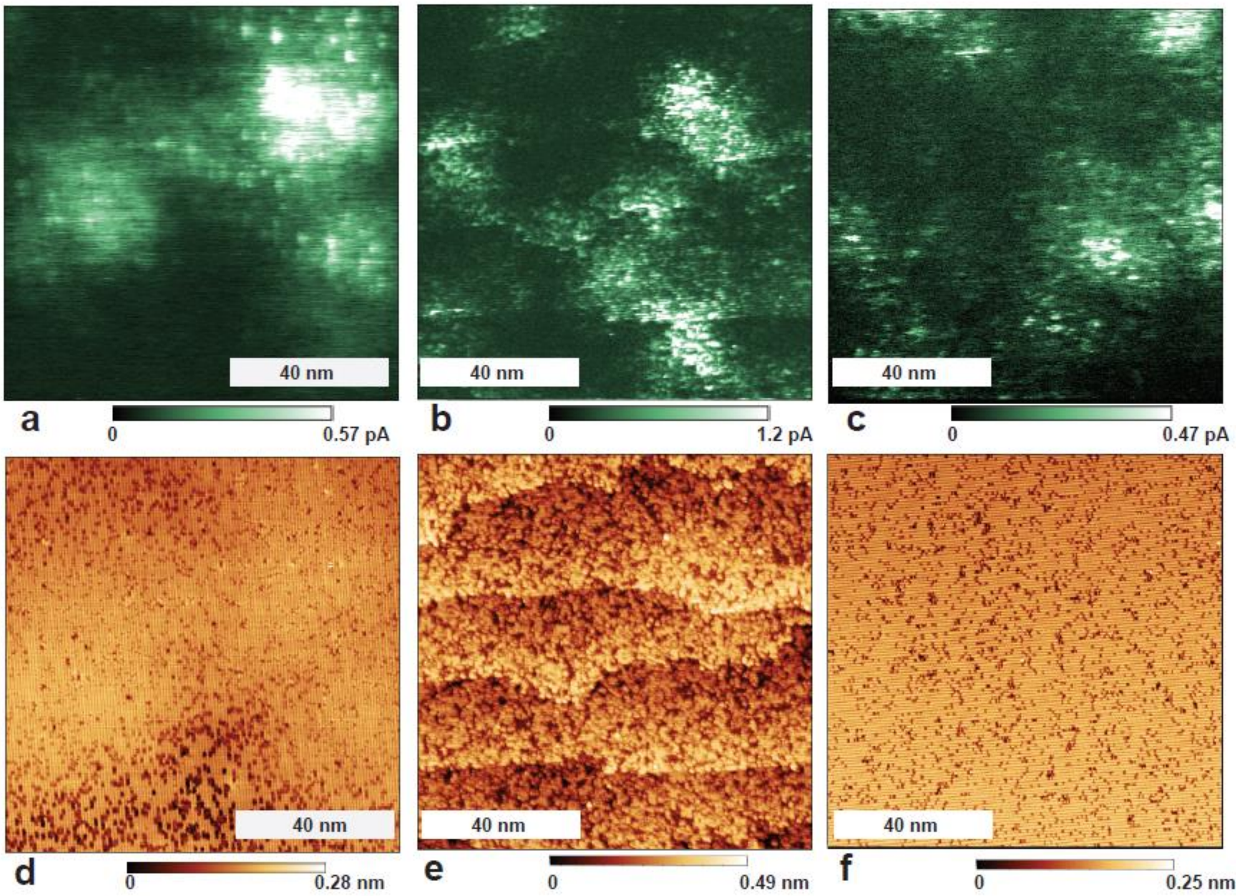

Figure #S2: Comparison of 100x100nm conduction AFM and STM image combinations taken on different samples taken from the same wafer. All images were acquired on samples from the same wafer under nominally identical measurement and preparation conditions. The top row displays conduction AFM images; the bottom row displays STM images at locations corresponding to the respective images in the top row. The STM images reveal that all three surfaces have different atomic scale structure and display different surface point defect densities. This variation is due to the fact that the three samples were flash annealed separately. The locations of the large current patches do not correlate with the terrace steps observed in the STM images.

The data set displayed in figure #S3 shows 6 images that were measured consecutively at the identical area, yet with different tip heights and different bias voltages. It is confirmed from all these repeated images that the influence of lateral tip-drift at 4.3K is insignificant and thus, it does not appear to play any role in c-AFM images. As can be seen, the contrast is similar for all the images except for Fig. #S3(c) where the current becomes undetectable. Details of the measurement conditions are given in the figure caption.

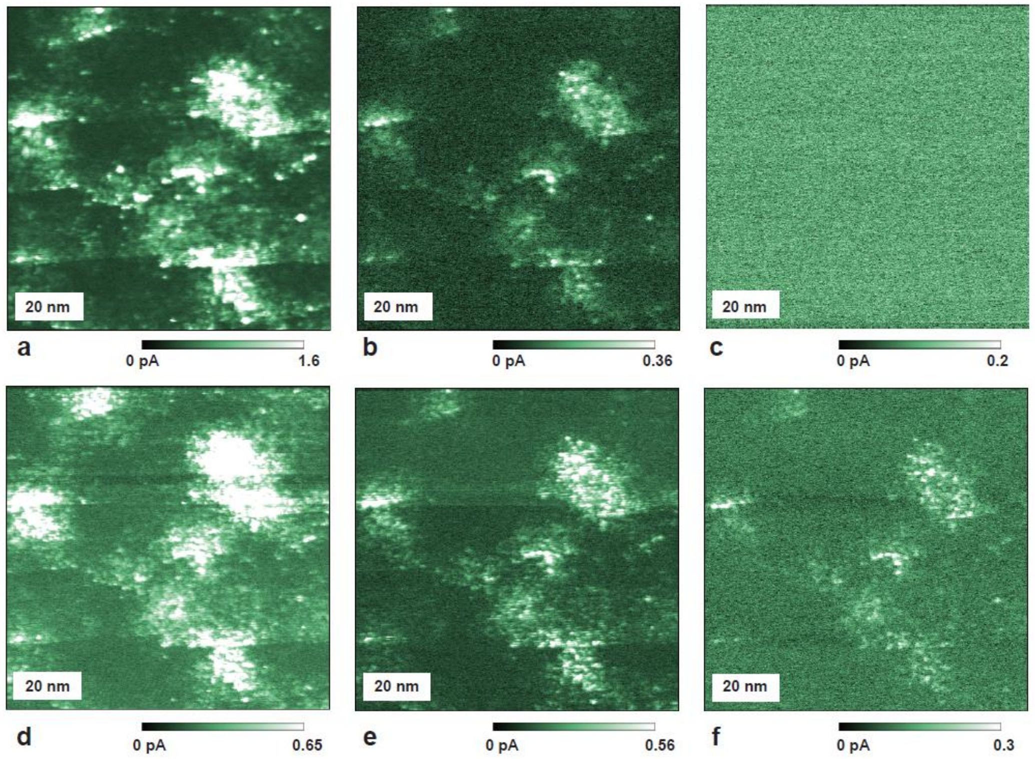

Figure # S3: c-AFM images at different gap and bias voltage. The data in panels (a), (b) & (c) were measured with constant tip-sample gap and a tip bias of 1.3V, 1.2V, and 1.1V, respectively. Panel (d), (e), and (f) where measured at a bias voltage of 1.2V with lower force feedback set-point that resemble an average ~1Å increase in the tip-sample gap successively from (d) to (f). Note that the panels (b) and (d) are two independent measurement and therefore taken at different tip-sample gap. The surface topography of these images is given in figure S2 (e). The data sets show how both, the presence of phosphorus atoms near the surface and the higher dangling bond density at step edges modulate the current.

2. Ångström-sized local current maxima

Figure #S4 displays data confirming the results shown in Fig. #2(b) through (d). Two sets of conduction AFM images were recorded at different locations under nominally identical conditions. As the experiments display in, i.e., Fig. #2(b), localized conduction is observed on a native oxide at 4.3K without light illumination. Figures #S4(a) and (d) clearly confirm the occurrence of highly localized current maxima which appear in larger, nm-range patch-like structures attributed to the P donor atoms. In contrast to the samples without native oxide, the density of localized maxima within the patches on the oxidized sample is significantly less dense than those observed on the bare silicon surface. The localized maxima indicate electronic states which are electronically coupled to a nearby P atom. We attribute the brightness (current) variations of these point-like states to different transition rates between these states and the P atoms and thus, to their physical distance to a nearby P donor state.

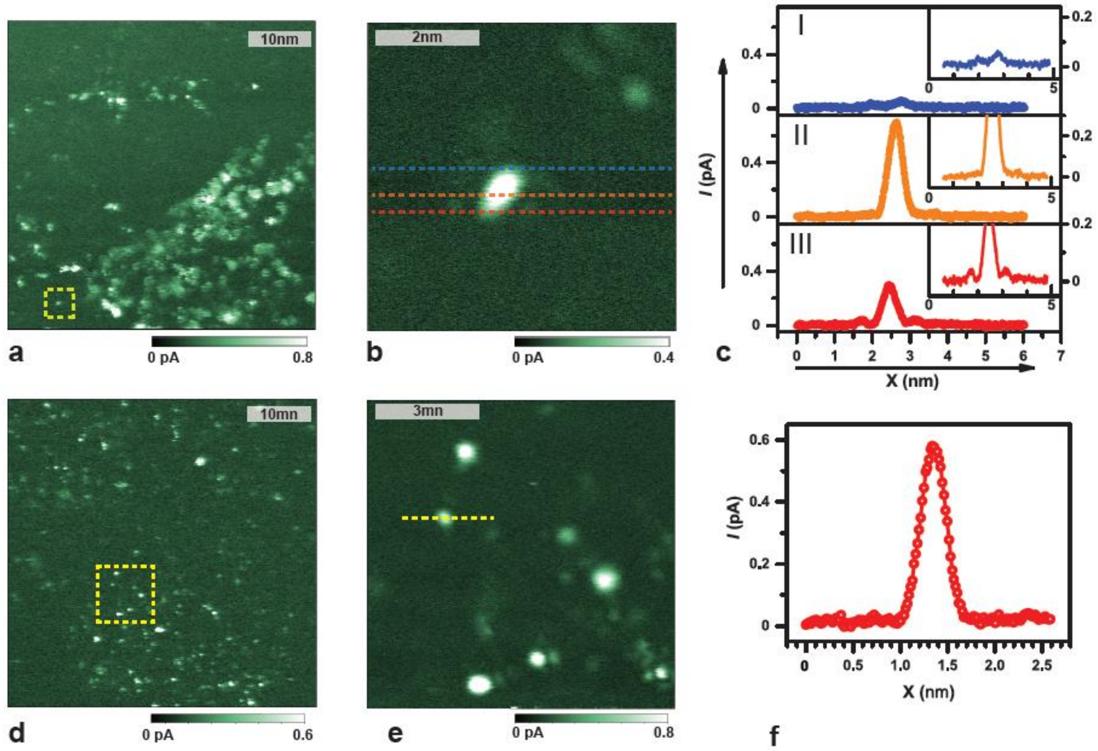

Figure #S4: Conduction AFM images of P doped ([P] ~ 10^{17}cm^{-3} to 10^{18}cm^{-3}) *c-Si surfaces with thin native oxide layers at 4.3K without light illumination. Panels (a) to (c) and (d) to (f) represent data sets collected at two different locations. Panel (a) and (d) both contain areas where patch-like clusters of local current maxima are observed while other areas show few localized current maxima. The local current maxima are due to highly localized electronic states in the oxide or at the c-Si oxide interface which are in electronic contact with nearby phosphorus atoms. They are attributed to silicon dangling bond states. Panels (b) and (e) are high resolution images taken from samples areas indicated by the yellow squares in panels (a) and (d), respectively. Panels (c) and (f) display line profiles of the local current maxima taken in the regions highlighted by the lines in panels (b) and (e), respectively. The widths of these local current maxima are indicative for the strong localization of dangling bond states.*

Panels (b) and (e) of Fig. #S3 are high-resolution images acquired at locations marked by yellow boxes in panels (a) and (d) respectively. Both panels (b) and (e) resolve individual current maxima of different shape and size. Similar to Fig. #1(d), panels (c) and (f) of Fig. #S3 show one dimensional line profiles which were recorded at these positions indicated by the lines in panel (b) and (e). The observed localization of these defects varies from ~3-5Å.

The very small localization and the discreteness of the fine structure observed within the P induced current patches is consistent with highly localized dangling bond [SS2], [SS3] states at the surface of the c-Si crystal or within the thin silicon dioxide network. The clustering of these highly localized electronic states within the patches is indicative that these states are connected electronically to a phosphorus donor state. Thus, while silicon dangling bonds likely exist at homogeneous densities throughout the observed sample areas, electric current is observed only through the highly localized surface states when they are connected to an adjacent P donor. As the P donor in closest proximity of the surface states is then connected to other P donors deeper in the bulk, the percolation paths that allow the observed currents are formed.

3. Phosphorus donor state accessibility by a Pt probe in a c-Si/SiO_2 system

Figure #S5 displays calculation results of the elastic tunneling rate from a Pt tip to a localized P donor state in a c-Si substrate as a function of the physical depth of the donor state from the surface for different tip-sample gap sizes using a static probe. The analytical model for these calculations was discussed by Zheng et. al., [SS4]. In this calculation the P donor was approximated by a finite spherical potential well width of 6nm diameter (approximately twice its Bohr radius) and a depth 0.055eV below the c-Si conduction band. With this configuration, the singly occupied ground state is 40.1meV below the conduction band, in agreement with literature values for P donor electron [SS5], [SS6]. Figure #S5 shows that a tunneling rate of one charge carrier per microsecond at 4.3K can be achieved for a donor depth of 12nm with a tip-sample gap of 3Å. This corresponds to a 160 fA tunneling current, well above the detection limit of the current preamplifier used in the experiments.

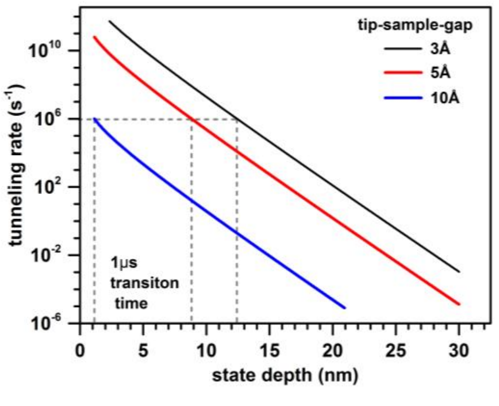

Figure #S5: Calculation of the electron tunneling rate from a Pt tip to a localized P donor electron state as a function of state depth for different tip-to-sample gap sizes. As expected, the tunneling rate decreases exponentially with depth. At a gap size of 3Å, electrons can tunnel as far as 12nm for a tunneling rate of 1e/µs, corresponding to a tunneling current 160fA.

3. Reproducibility and uniqueness of I-V curves

While current-voltage (I-V) curves measured with conduction AFM at different sample locations and conditions fall into four characteristic groups as discussed in the main text, all conduction AFM experiments have shown excellent reproducibility of I-V curves when measurements were repeated at identical sample locations and nominal experimental conditions. In order to demonstrate this reproducibility, repeated experiment were conducted at local current maxima. Figure S6 displays the results for repeated I-V curves conducted at two different locations. For each of these locations, only very small differences between each of the measured I-V curves were observed. We attribute the small differences that are observed to the finite thermal drift of the cantilever probe as it moves slowly away from the selected defect state. Defect-1 and defect-2 represent two independent current maxima at different locations pertaining to examples if I-V functions represented by the qualitatively similar data in Fig. #5(c) and (a), respectively. Each experiment was repeated seven times.

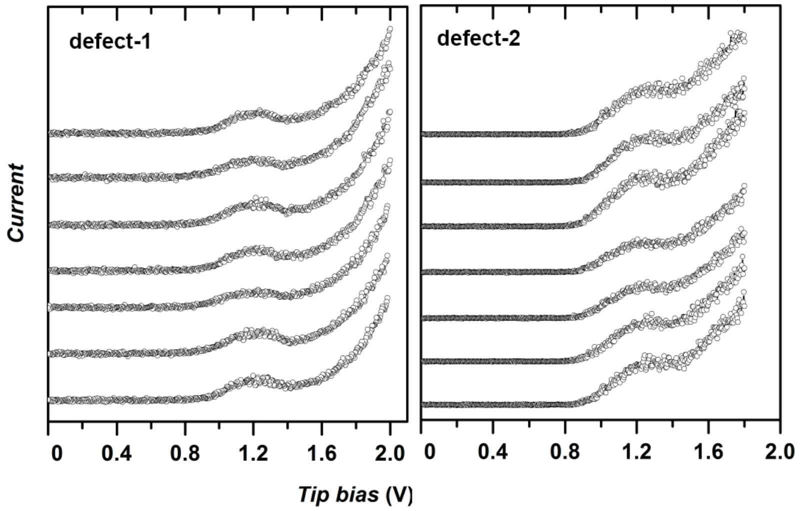

Figure #S6: Repetition of current-voltage (I-V) measurements using conduction AFM for two randomly chosen surface locations 'Defect-1' and 'Defect-2'. While the two locations display different I-V characteristics, they display very reproducible characteristics for each location. Each curve has identical vertical axes. Since the curves are offset along the vertical axis, no vertical axis level is printed in the plot.

Since dangling bond states exist at the interface between the c-Si and silicon dioxide as well as within the silicon dioxide, they are all unique due to the randomness of their individual microscopic environment (the continuous random network of the amorphous silicon dioxide). This is the reason that one would expect to see equally random variations in the I-V curves obtained on different dangling bond states. This is further supported by the fit of the distribution of measured energies of the dangling bond states with published data, as shown in Fig. S4(h).

Figure #S7 displays an array of I-V curves recorded with the conduction AFM in darkness and at T = 4.3K at various arbitrary positions across an oxidized c-Si surface. The displayed data sets represent a very small subset of all measured I-V curves which are additional examples for the four qualitatively different

I-V curves that have been observed. Each row in the array of Fig. #S7 displays several qualitatively identical examples. The first row shows single flat plateau I-V curves, the second row shows single-plateau I-V curves with negative slope, the third row displays monotonous diode-like I-V curves, and in the fourth row another single double-plateau I-V curves is presented. Comparing the data sets within each row shows that within each category, there are still significant quantitative differences. For instance, for the single flat plateau curves displayed in the top row of Fig. #S7, the onset and endpoints as well as the width of the plateaus are different for each data set. Similarly, the magnitudes of currents vary from a few hundreds of fA to a few pA. Comparing the second row data sets (single plateau curves with negative slope at the plateau), each plateau occurs at different current magnitude and the slope of each plateau differs from each other. Also, the curves in the third row qualitatively look like with diodes but both the magnitude of current and the turn on voltage different from each other. The last row displays double plateau curve. These were observed only 30 times among the more than 800 measured I-V curves.

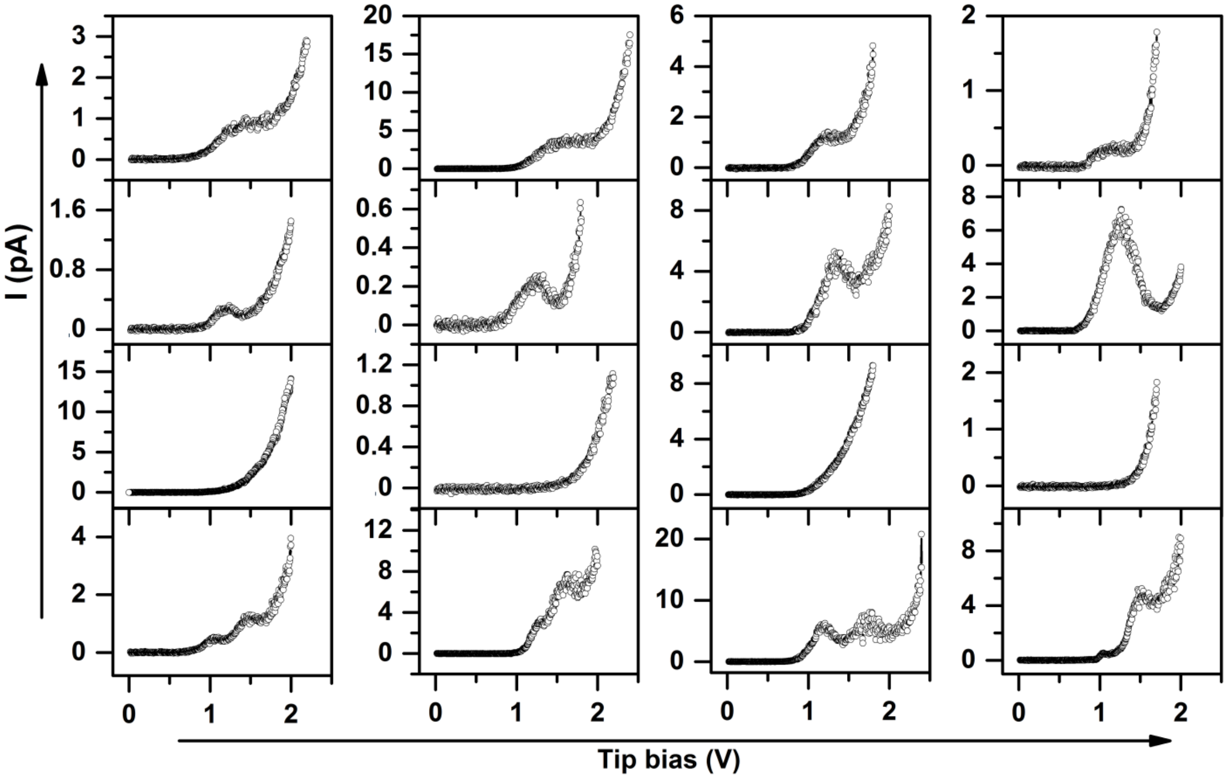

Figure #S7: Plots of I-V curves measured under nominally identical conditions at various locations of one sample. Each column of the figure represents four measurements performed at locations within a proximity of a few hundred micrometers. While all measured I-V functions are quantitatively different, measurements conducted at each location could be categorized into four qualitatively different sets of curves as discussed in the main text. The double plateau curve shown in the fourth row has been consistently rare (< 4% of all measurements).

Figure #S8 displays IV curves from three different qualitative IV curve categories, measured at both positive and negative polarity of the tip-sample bias. For each of the displayed IV curves, the current in the forward direction (positive polarity) is significantly higher compared to the current with reversed biased (negative polarity).

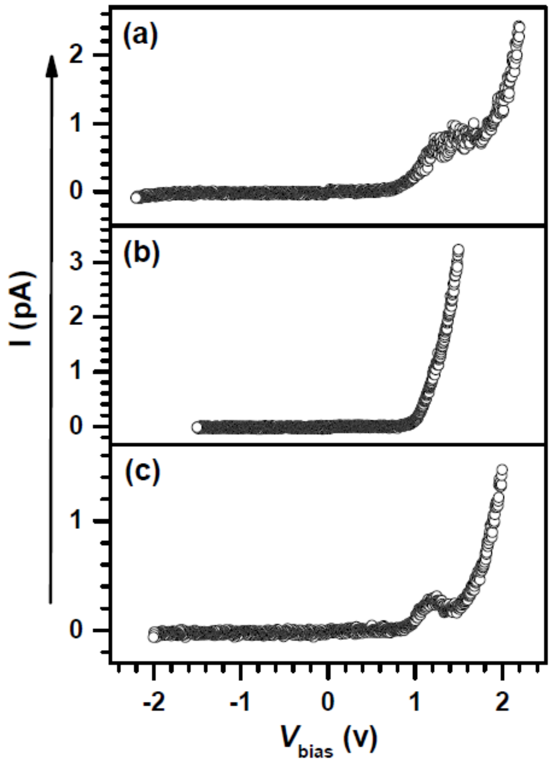

Fig #S8 : I-V curves with both positive and negative bias for surface locations displaying a flat-plateau, a Schottky behavior, and a shoulder-plateau, respectively. In all cases, the forward bias produced significantly higher current compared to negative bias where little to no current was observed. In general, the IV curves are always diode like.

4. Model hypothesis for the observed “single-flat plateau” I-V characteristics

Figure #S9 displays a band diagram of the P-doped c-Si/SiO_2 interface in presence of a nearby metal probe placed at a tip-sample gap distance (d= ~3 Å) above a thin SiO_2 layer with thickness (b= 2-3 Å [SS7, SS8]) on a phosphorus doped silicon substrate. It is assumed that electrons can percolate to a back contact, even at low temperatures, as the substrate is highly doped with phosphorus donors. Within the c-Si, the Fermi energy will be above the donor energy level, yet below the conduction band. The AFM probe tip behaves like a bulk metal. When the bias applied to the tip is positive and sufficiently large in magnitude such that the Fermi energy in the probe is below the energy of an interface defect state, a continuous current is established consisting of transitions from the P donor into the interface defect followed by a transition from the interface defect into the metal. Note that when the bottleneck transition in this cascade is the P-donor to defect transition, a change of the probe’s Fermi energy will not cause a change of the overall current. Thus, a flat plateau will be observed in the I-V curves within a positive bias range.

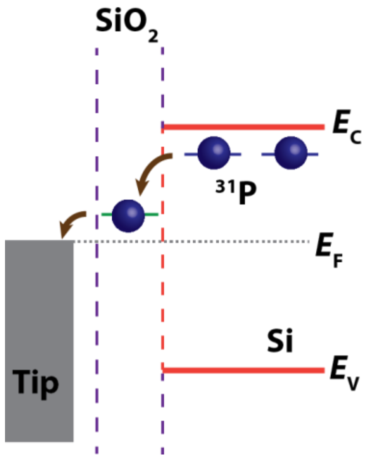

Figure #S9: Energy diagram of Si/SiO_2 interface. E_V and E_C are the valance- and conduction-band energies of c-Si which are separated by 1.1eV. Donor electrons occupying the phosphorus states (blue spheres) are 40meV below the conduction-band energy. At 4.3K the donor electrons are frozen into the donor states. The Brown arrows represent the percolation of electrons from the back contact to the metal probe. The green line indicates the localized electronic state that is either in the silicon dioxide layer or at the interface between silicon dioxide and the c-Si substrate.

5. IV curves without native oxide silicon surface.

Figure #S10 displays IV curves measured at different locations on a silicon surface without any oxide. The average dangling bond density on such an unterminated surface is significantly higher compared to a silicon surface with oxide layer. It is therefore conceivable that the dangling bond states couple to each other and many current percolation paths from the P-donor state to surface dangling bond states are possible for any location across the surface, possibly even involving delocalized surface state. This leads to an overall increased current which, even if detected locally with a scanning probe tip, essentially reflects macroscopic bulk conductivity. The IV characteristics shown in Fig. S10 supports this picture: At any position, the IV functions resemble a Schottky-diode characteristics, never displaying the plateau features observed for oxidized surfaces. This is consistent with the hypothesis that the plateau features are caused by microscopic bottleneck transitions due to charge percolation through a few well-defined localized electronic states.

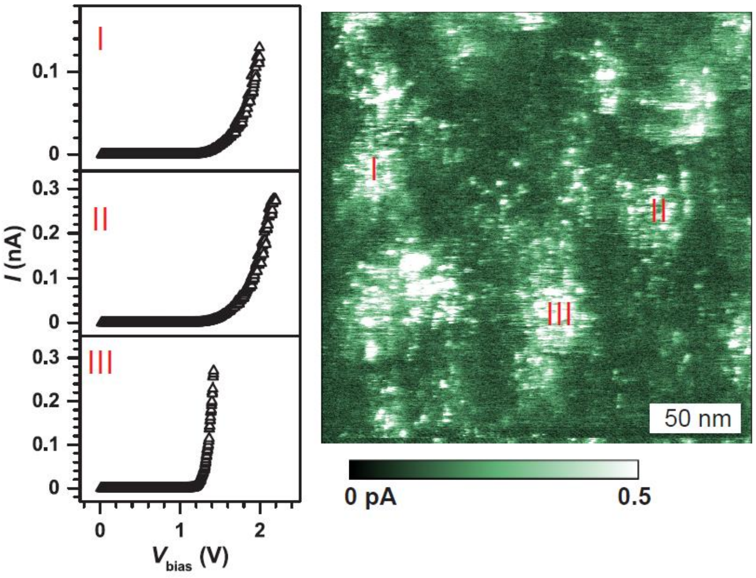

Figure #S10: Panels (I), (II) and (III) represent I-V curves measured at three different locations indicated in the c-AFM image that was taken on a silicon surface without oxide at tip bias 1.2V. The dangling bond density on such a surface is very high for any given location and the current is significantly higher compared to single point dangling bond IV curves measured at silicon surfaces with grown oxides.

6. Bigger surface scan of the figure 3:

Figure #S11 is a larger are surface scan image corresponding to Fig. #3(c) and (d). Panel (a) and (b) of the Fig. #S11 is the identical image of panel (c) and (d) of Fig. #3. Panel (c) of Fig. #S11 displays the larger area STM image and the white box represents the area where panel (a) was measured. Panel (d) is the corresponding c-AFM image taken at the identical area as panel (c) and the marked box represents the area where panel (b) was measured independently. The effect of step edges in panel (d) is visible but in poor contrast due to overall higher surface point defect density.

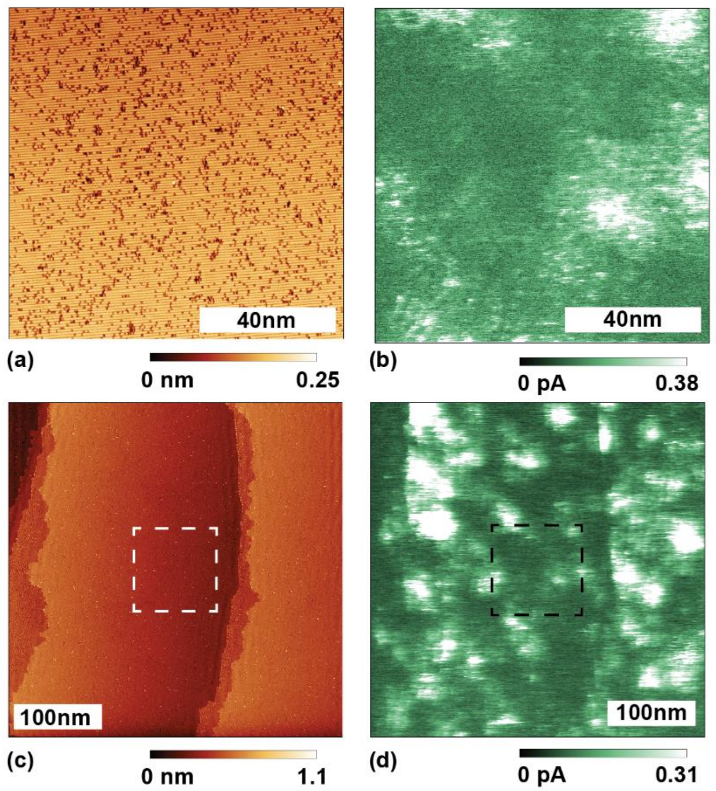

Figure #S11: *STM and c-AFM image of a P-doped c-Si crystal surface. Panel (a) is the STM image taken independently at the location shown as box in panel (c). Panel (b) and (d) are c-AFM images taken at 1.2 V tip bias. Panel (b) is the c-AFM image independently measured at the location marked as box in panel (d).*

References

[S1] Masayasu, N. et al. Origin of type-*c* defects on the Si(100)-(2×1) surface. *Phys. Rev. B* **65**, 161302 (2002).

[S2] Petters J. L. et al. Tunnel coupled dangling bond structures on hydrogen terminated silicon surfaces. *J. Chem. Phys.***134,** 064712 (2011).

[S3] Hoehne, F. et al. Electrically detected electron-spin-echo envelope modulation: A highly sensitive technique for resolving complex interface structures. *Phys. Rev. Lett.* **106** 196101 (2011).

[S4] Zheng, N., Williams, C. C., Mishchenko, E. G., and Bussmann, E. A.Three-dimensional model of single-electron tunneling between a conductive probe and a localized electronic state in a dielectric. *J. Appl. Phys.* **101**, 093702 (2007).

[S5] Pica G. et al.Hyperfine stark effect of shallow donors in silicon. *Phys. Rev. B* **90**, 195204 (2014).

[S6] Ramdas A. K. and Rodriguez, S. Spectroscopy of the solid-state analogues of the hydrogen atom: donors and acceptors in semiconductors, *Rep. Prog. Phys.* **44**, 1297 (1981).

[S7] Morita M. et al. Growth of native oxide on a silicon surface. *J.Appl. Phys.* **68,** 1272–1281 (1990).

[S8] Uemura, S. et al. In situ observation of native oxide growth on a Si(100) surface using grazing incidence x-ray reflectivity and fourier transform infrared spectrometer. *Jpn. J. Appl. Phys.* **40**, 5312 (2001).
